# White matter tracts involved in complex regional pain syndrome after subcortical stroke

**DOI:** 10.3389/fneur.2025.1699775

**Published:** 2025-11-24

**Authors:** Seungwoo Cha, ByeongChang Jeong, Hee-Mun Cho, Won Kee Chang, Nam-Jong Paik, Won-Seok Kim, Cheol E. Han

**Affiliations:** 1 Department of Rehabilitation Medicine, Asan Medical Center, University of Ulsan College of Medicine, Seoul, Republic of Korea; 2 Department of Electronics and Information Engineering, Korea University, Sejong, Republic of Korea; 3 Interdisciplinary Graduate Program for Artificial Intelligence Smart Convergence Technology, Korea University, Sejong, Republic of Korea; 4 Department of Rehabilitation Medicine, Seoul National University College of Medicine, Seoul National University Bundang Hospital, Seongnam, Republic of Korea; 5 Division of Smart Energy Convergence Engineering, Korea University, Sejong, Republic of Korea

**Keywords:** stroke, subcortical, white matter tract, complex regional pain syndrome, atlas-based lesion overlapping analysis

## Abstract

Post-stroke complex regional pain syndrome (CRPS) is a challenging complication that impairs recovery during stroke rehabilitation, particularly during the subacute phase. Despite its clinical significance, the neural substrates underlying post-stroke CRPS, specifically following subcortical stroke, remain unclear. This retrospective observational study included 40 patients with first-ever subcortical stroke diagnosed with CRPS via a three-phase bone scan, and 40 propensity score-matched controls without CRPS. White matter tract involvement was analyzed using atlas-based lesion mapping and voxel-based lesion overlap analysis in patients with available clinical scores and imaging findings (magnetic resonance imaging or computed tomography). Between-group comparisons of white matter tract involvement were conducted with false discovery rate (FDR) correction. Clinical characteristics were similar between groups, except for fewer CRPS patients with a shoulder flexor manual muscle test score ≥3. Lesion overlap with the cingulum in the cingulate gyrus was significantly greater in the CRPS group (*F* = 5.197, FDR-adjusted *p* = 0.040). Although the forceps minor showed marginal significance before correction, it was non-significant after adjustment. These findings raise the possibility that the cingulate cortex, particularly the cingulum, may contribute to post-stroke CRPS pathophysiology. However, further confirmation in larger prospective studies is needed.

## 1 Introduction

Post-stroke complex regional pain syndrome (CRPS) is a notable and potentially debilitating complication of stroke. Incidence estimates vary considerably across studies; however, a meta-analysis of 21 studies reported a pooled rate of approximately 32% among stroke survivors, predominantly Type I cases (reflex sympathetic dystrophy) without identifiable peripheral nerve injury (1). CRPS occurs more frequently in females and those with left-sided hemiparesis. Post-stroke CRPS typically emerges during the subacute phase, approximately 1 month after stroke onset, thereby complicating rehabilitation (2). Consequently, early recognition and appropriate management are critical to optimize rehabilitation planning and functional recovery.

One study identified the caudate nucleus, putamen, and white matter structures, particularly the corticospinal tract (CST), as key regions associated with post-stroke CRPS, although it included patients with cortical and subcortical stroke and lacked CRPS-specific diagnostic confirmation (3). However, evidence indicates that subcortical strokes may involve distinct pathophysiological mechanisms compared to cortical strokes, disrupting network-level neurophysiology and inducing cortical reorganization that supports functional recovery (4, 5). Therefore, distinguishing subcortical stroke from cortical stroke is crucial for understanding the neural basis of CRPS and developing individualized rehabilitation strategies tailored to lesion location and underlying neural network disruption. Similarly, identifying subcortical neural correlates, as shown in a previous study on unilateral spatial neglect (6), may offer valuable insights into the pathophysiology of post-stroke CRPS in patients without cortical involvement.

Therefore, this study aimed to investigate specific white matter tracts as potential neural substrates of CRPS in patients with subcortical stroke using atlas-based tract mapping and voxel-based lesion overlap analysis. Furthermore, only patients objectively diagnosed with CRPS via three-phase bone scan were included.

## 2 Materials and methods

### 2.1 Participants

This retrospective observational study included patients admitted for stroke who underwent a three-phase bone scan at Seoul National University Bundang Hospital between March 2003 and March 2021. Of 492 confirmed stroke cases reviewed, 236 were excluded because the scan was not for CRPS evaluation or yielded negative results. Additional exclusions included prior stroke, cortical, brainstem, or cerebellar stroke, absence of brain imaging [magnetic resonance imaging (MRI) or computed tomography (CT)], and missing data. The final CRPS group comprised 40 patients with first-ever subcortical stroke and scan-confirmed CRPS.

**Figure 1:**
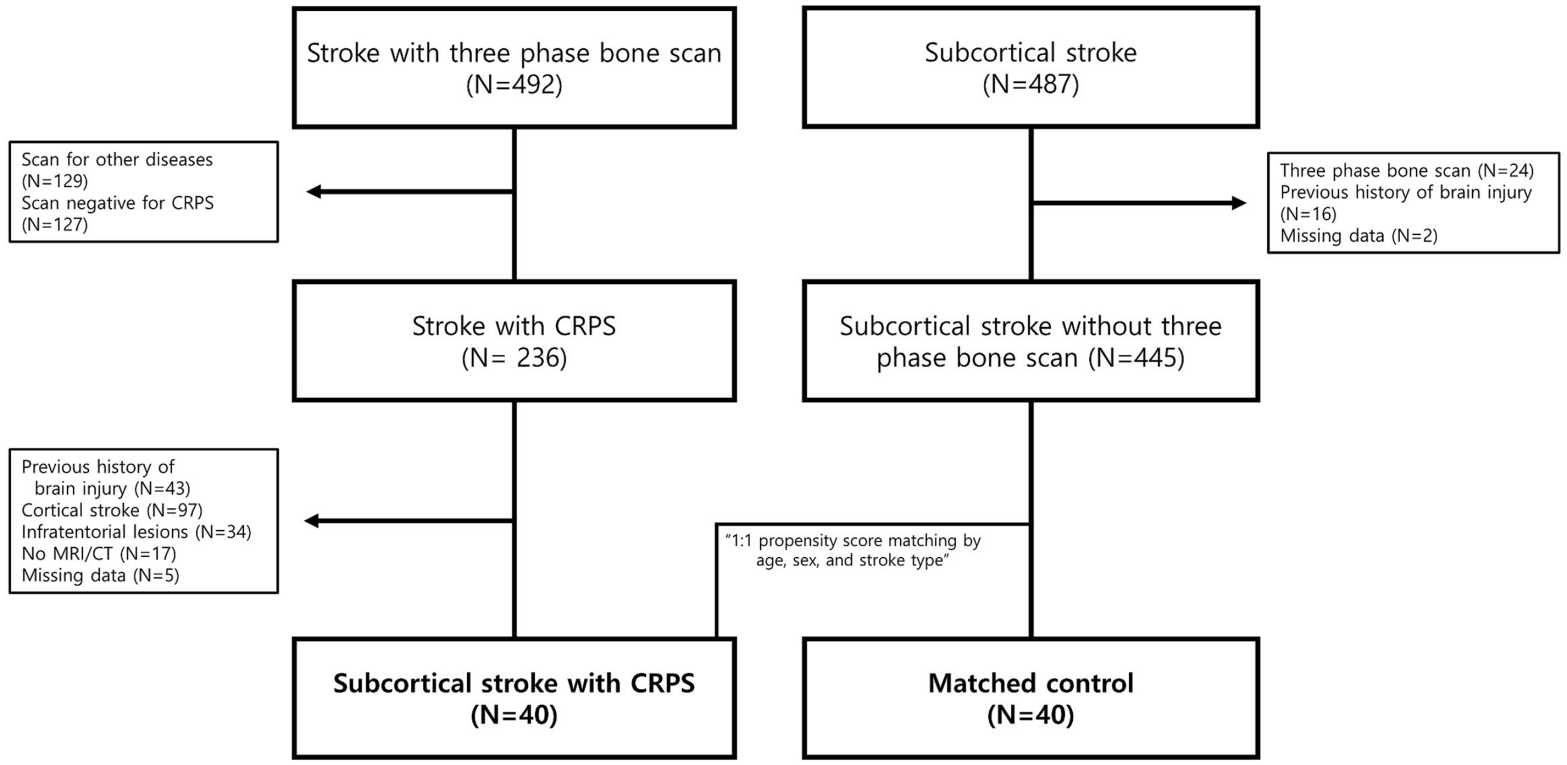
Patient flow.

For the control group, 487 patients diagnosed with subcortical stroke were identified during the same period who did not undergo a three-phase bone scan. Patients were excluded if they had a history of stroke, had undergone a bone scan, or lacked clinical data, resulting in a total of 445 eligible candidates. Forty patients were then selected from this pool using 1:1 propensity score matching based on age, sex, and stroke type to serve as the control group (Figure 1).

Clinical data collected for each patient included the initial National Institutes of Health Stroke Scale (NIHSS) score, Modified Barthel Index (MBI), Fugl-Meyer Assessment for Upper Extremity (FMA-UE), shoulder flexor strength assessed by manual muscle testing (MMT), and spasticity measured using the Modified Ashworth Scale. The intervals from stroke onset to CT or MRI, and for the CRPS group, to the three-phase bone scan were recorded.

This study was approved by the Seoul National University Bundang Hospital Institutional Review Board, which waived the need for informed consent (IRB No. B-1706-401-102).

### 2.2 Acquisition and preparing of imaging data

Neuroimaging data were collected retrospectively from 80 patients as part of their clinical evaluation at Seoul National University Bundang Hospital. The dataset included 50 fluid-attenuated inversion recovery (FLAIR) scans, 27 CT scans, and three diffusion-weighted imaging (DWI) scans. Preprocessing was modality-specific to ensure consistent anatomical alignment across participants. For participants who underwent both T1-weighted and lesion-sensitive MRI (FLAIR or DWI), the lesion-sensitive image was linearly co-registered to the T1-weighted structural image. T1 images were subsequently nonlinearly normalized to the International Consortium for Brain Mapping (ICBM) standard brain template for East Asian populations using Statistical Parametric Mapping (SPM12) (7). The resulting transformation parameters were applied to the co-registered lesion-sensitive image, which was resliced into template space. This two-step approach enabled precise normalization while preserving lesion localization.

For participants with CT images only, normalization was performed using the Clinical Toolbox extension of SPM12 (8), which incorporates a CT-specific template and optimized normalization pipeline. Each CT scan was co-registered to the standard CT template provided by the toolbox and then transformed into ICBM space. This method allows for effective spatial normalization without an MRI-based structural reference.

Lesions were manually delineated on the normalized images using MRIcron software (9) by two authors (SC and H-MC) and subsequently supervised by N-JP and W-SK, all are physiatrists with expertise in neuroanatomy. The raters had prior experience in lesion delineation and have contributed to previous lesion-symptom analysis studies (6, 10). All lesion masks were generated in a standardized space for subsequent analyses. To enable group-level comparisons, lesions located in the right hemisphere were horizontally flipped to the left side.

### 2.3 Analysis of white matter tract involvement

We examined the extent to which individual lesions disrupted major white matter tracts in the left hemisphere following previous studies (6, 10). Using the probabilistic white matter atlas developed by Johns Hopkins University, we selected 11 anatomically and clinically relevant tracts (11, 12): the anterior thalamic radiation, cingulum in the cingulate gyrus (CGC), cingulum in the hippocampal region, CST, forceps major, forceps minor (FMIN), inferior fronto-occipital fasciculus, inferior longitudinal fasciculus, superior longitudinal fasciculus, temporal projections of the superior longitudinal fasciculus, and the uncinate fasciculus.

For each participant, we calculated the overlap volume between the binarized lesion mask and each tract by counting the number of intersecting voxels. To compare tract involvement between the CRPS and control groups, we performed a permutation-based analysis of covariance (ANCOVA), adjusting for age and gender (13). Given the small sample size and non-normally distributed overlap volumes, non-parametric permutation testing with 50,000 iterations was used (14, 15). To correct for multiple comparisons across the 11 tracts, we applied the false discovery rate (FDR) procedure (16). Statistical significance was set at *p* < 0.05.

Statistical analyses were performed using custom MATLAB scripts (R2024b, MathWorks) and the LinStat toolbox (2006b) (17).

### 2.4 Lesion overlapping analysis

To provide a spatial overview of the lesion distribution and its relationship with white matter tracts, group-level lesion overlaps were generated for the CRPS and control groups by aggregating the normalized lesion masks of all the individuals in each group. To highlight the lesion patterns specific to CRPS, the overlap map of the control group was subtracted from that of the CRPS group, yielding a symptom-related lesion map. We overlaid the map with all 11 predefined white matter tracts, but only tracts that spatially overlapped with the lesions were displayed in the visualization.

Image processing and overlays were performed using the FMRIB Software Library (FSL, version 5.0.9) (18), BrainNet Viewer for surface-based visualization (19), and voxel lesion symptom mapping (VLSM, version 2.55; https://aphasialab.org/vlsm/) for voxel-level spatial maps. Although VLSM statistical analysis was not conducted, the toolbox was used to generate structured visual representations.

To assess the distributional differences in lesion volume overlaps across groups, the empirical cumulative distribution function (ECDF) was analyzed, providing a non-parametric view of the cumulative frequency of observed data values, which was generated using MATLAB (R2024b, MathWorks).

### 2.5 Statistical analysis for clinical characteristics

Continuous variables were tested for normality. Normally distributed data were expressed as mean ± standard deviation and compared using Student’s *t*-test, whereas non-normally distributed data were presented as median and interquartile range, and group comparisons were performed using non-parametric tests. Categorical variables were compared using the chi-squared test. For multiple comparisons, FDR correction was applied using the Benjamini–Hochberg procedure to adjust the *p*-values. Statistical analyses of the clinical characteristics were conducted using R software (version 4.4.2, Vienna, Austria).

## 3 Results

### 3.1 Clinical characteristics

There were no significant differences in most clinical characteristics between the CRPS and control groups; however, the proportion of participants with shoulder flexor MMT ≥3 was significantly lower in the CRPS group (Table 1).

**Table 1:** Clinical characteristics.

Variables	CRPS (*n* = 40)	Control (*n* = 40)	*p*-value
Age	64.4 ± 11.4	64.4 ± 13.8	0.97
Male	19 (47.5%)	17 (42.5%)	0.65
Lesion side			0.82
Right	16 (40.0%)	18 (45.0%)	
Left	24 (60.0%)	22 (55.0%)	
Stroke type			0.50
Infarction	19 (47.5%)	23 (57.5%)	
Hemorrhage	21 (52.5%)	17 (42.5%)	
Initial NIHSS	11.1 ± 6.1 (*n* = 32)	9.9 ± 6.1 (*n* = 36)	0.43
K-MMSE	15.0 ± 9.9 (*n* = 37)	19.3 ± 9.6 (*n* = 38)	0.06
MBI	22.0 ± 18.4 (*n* = 38)	30.5 ± 22.5 (*n* = 39)	0.07
FMA-UE	15.4 ± 17.8 (*n* = 34)	20.0 ± 20.4 (*n* = 40)	0.31
Shoulder flexor strength			0.001^*^
≥3	2 (8.0%)	15 (48.4%)	
<3	23 (92.0%)	16 (51.6%)	
Spasticity	5 (22.7%) (*n* = 22)	2 (11.1%) (*n* = 18)	0.43
Imaging			1.00
MRI	26 (65.0%)	27 (67.5%)	
CT	14 (35.0%)	13 (32.5%)	
Lesion volume (mL)	18,432 [4,906…58,718]	14,192 [4,279…37,886]	0.32
Onset to MRI/CT, days	0.5 [0…5.75]	0.5 [0…5]	0.86
Onset to bone scan, days	61 [36…123.25]	n/a	n/a

CRPS, complex regional pain syndrome; NIHSS, National Institutes of Health Stroke Scale; K-MMSE, Korean version of the Mini-Mental State Examination; MBI, Modified Barthel Index; FMA-UE, Fugl-Meyer Assessment for Upper Extremity; MRI, magnetic resonance imaging; CT, computed tomography; n/a, not applicable. ^*^p-value <0.05.

### 3.2 White matter tract analysis

**Figure 2:**
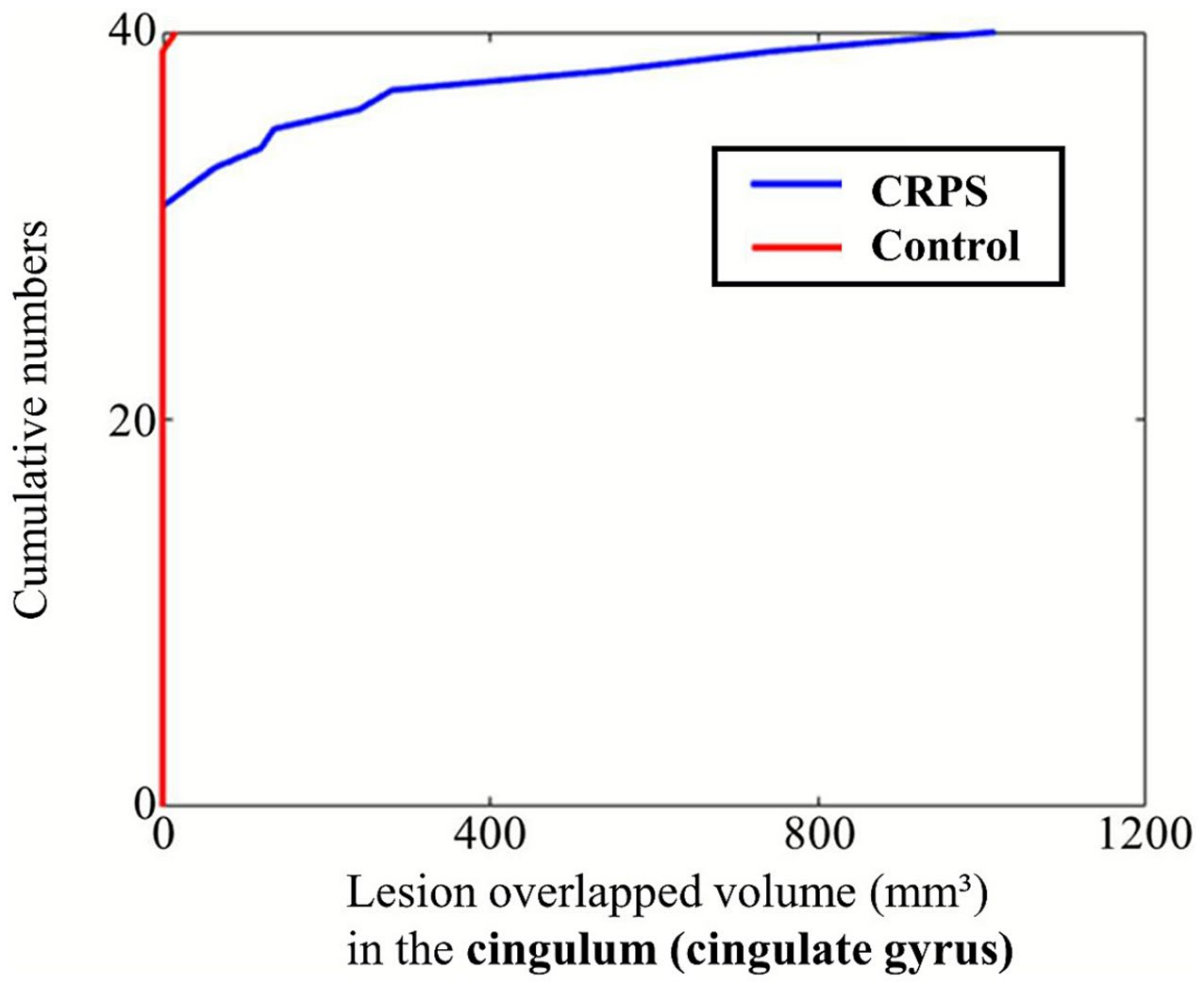
ECDF plot of lesion overlap volume with the cingulum (cingulate gyrus) in the CRPS and control groups. The ECDF represents the cumulative probability that the lesion overlap volume is less than or equal to a given value. A leftward shift of the curve indicates smaller overlap volumes, whereas a rightward shift reflects larger overlaps. The plot demonstrates group-wise differences in lesion involvement of the cingulum. CRPS, complex regional pain syndrome; ECDF, empirical cumulative distribution function.

White matter tract analysis revealed significantly greater lesion overlap in the CGC in the CRPS group compared with controls (*F* = 5.197, FDR-adjusted *p* = 0.040). Although the median lesion overlap was 0 in both groups, the ECDF plot demonstrated a clear divergence between the groups, indicating a higher overall burden in the CRPS group (Figure 2). Lesion overlap in the FMIN showed a marginal difference before correction (*F* = 3.595, uncorrected *p* = 0.053); however, this difference was not significant after FDR correction. No significant differences were found between the groups in the CST, anterior thalamic radiation, or other major tracts after FDR correction (Table 2). In subgroup analyses according to stroke subtype (infarction and hemorrhage), there were no significant differences in the CGC or FMIN between the CRPS and control groups (Supplementary Tables 1, 2). Functional outcomes, including the FMA-UE, shoulder flexor MMT, and MBI, were not significantly associated with lesion overlap.

**Table 2:** Overlap (mm^3^) of the lesions in each group with the major white matter tracts.

White matter tractography atlas	CRPS	Control	*F*-statistics	Uncorrected *p*-value	FDR-adjusted *p*-value
Anterior thalamic radiation	288 [8…1988]	56 [0…880]	1.804	0.18	0.45
Corticospinal tract	720 [72…1,440]	580 [88…1,244]	0.294	0.59	0.67
Cingulum (cingulate gyrus)	0 [0…8]	0 [0…0]	5.255	0.003^*^	0.033^*^
Cingulum (hippocampus)	0 [0…0]	0 [0…0]	0.844	0.65	0.67
Forceps major	0 [0…0]	0 [0…0]	2.407	0.16	0.45
Forceps minor	0 [0…0]	0 [0…0]	3.580	0.052	0.29
Inferior fronto-occipital fasciculus	624 [0…2088]	520 [0…1,672]	1.421	0.23	0.45
Inferior longitudinal fasciculus	0 [0…904]	0 [0…264]	0.680	0.41	0.57
Superior longitudinal fasciculus	180 [0…1,628]	0 [0…784]	1.145	0.29	0.45
Uncinate fasciculus	20 [0…400]	0 [0…212]	1.357	0.25	0.45
Superior longitudinal fasciculus	0 [0…0]	0 [0…0]	0.654	0.67	0.67

CRPS, complex regional pain syndrome; FDR, false discovery rate. ^*^p-value <0.05.

### 3.3 Symptom-related lesion distribution

**Figure 3:**
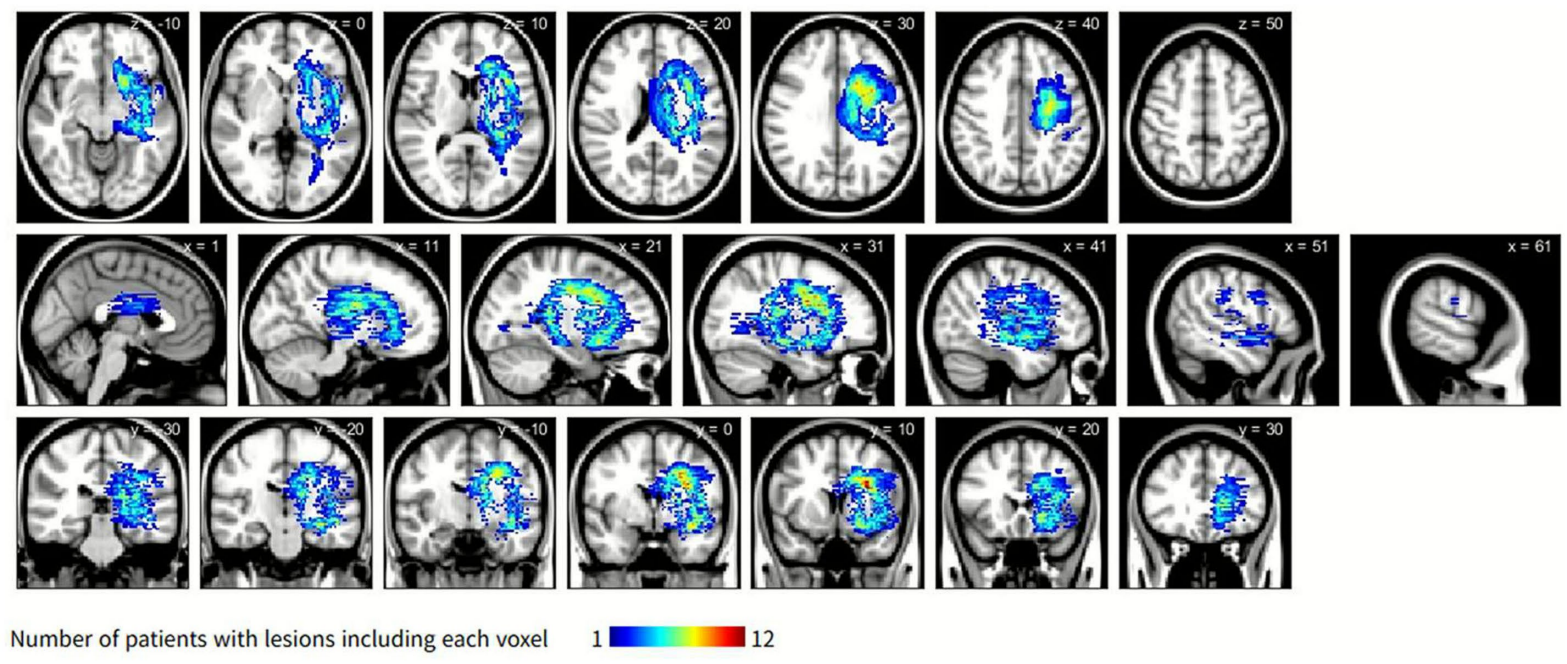
Symptom-related lesions obtained by subtracting the lesion distribution of the control group from that of the CRPS group. The color scale indicates the number of CRPS participants with overlapping lesions, with blue representing fewer overlaps and yellow to red representing progressively greater overlaps. Overlaps are primarily located in the caudate nucleus, putamen, and corona radiata. CRPS, complex regional pain syndrome.

The symptom-related lesion distribution obtained by subtracting the lesion maps of the control group from those of the CRPS group is shown in Figure 3. The lesion clusters were predominantly observed in the caudate nucleus, putamen, and corona radiata.

### 3.4 Tract overlap visualization

**Figure 4:**
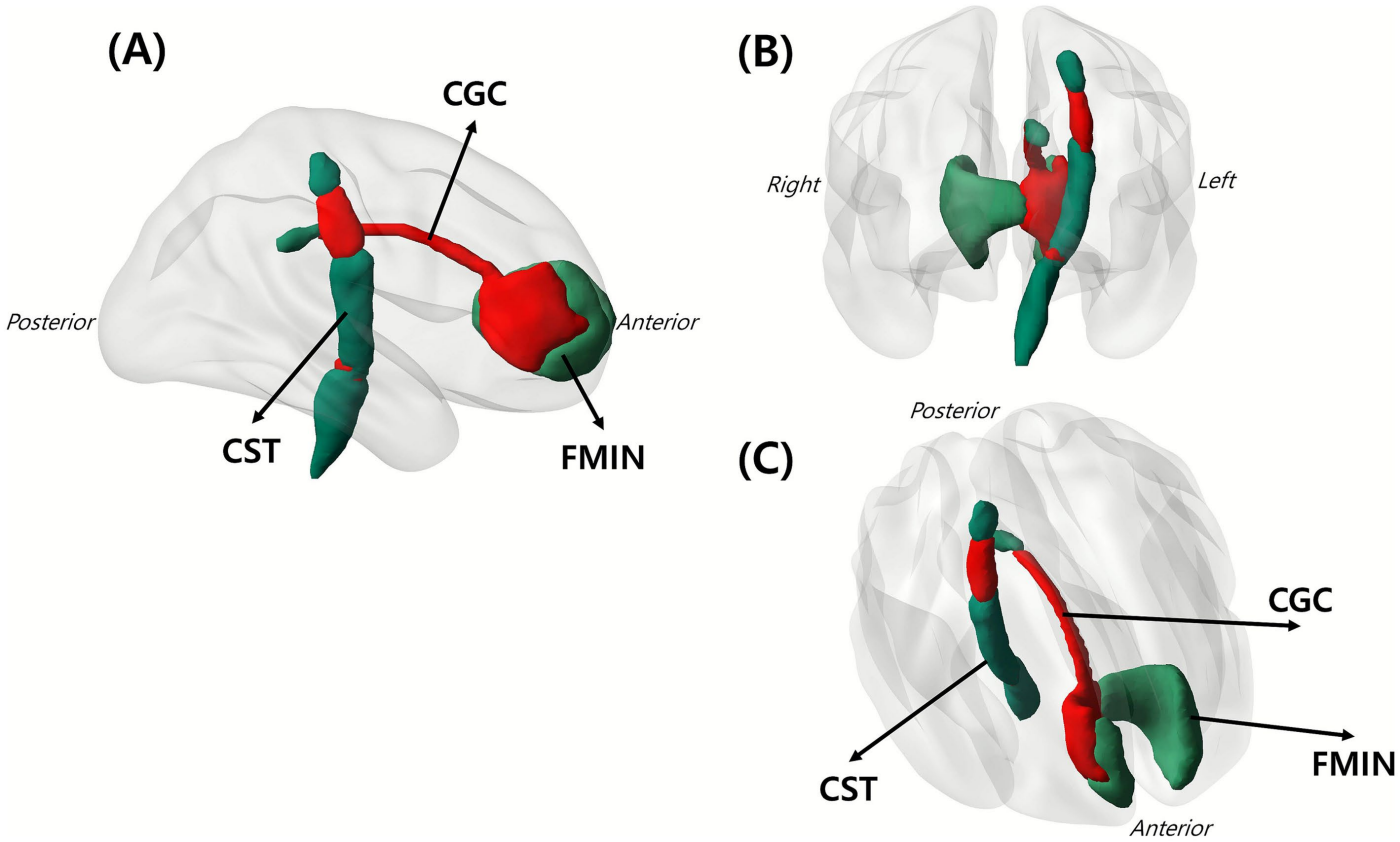
Overlap of white matter tracts with the identified symptom-related lesions. Red area indicates the spatial intersection between lesions and major white matter tracts, demonstrating where CRPS-related lesions involved specific tracts. Shown are **(A)** sagittal view, **(B)** coronal view, and **(C)** lateral oblique view of the left hemisphere. The figure involves three key tracts: CGC, CST, and FMIN. CGC, cingulum in the cingulate gyrus; CST, corticospinal tract; FMIN, forceps minor.

Figure 4 shows that symptom-based lesions overlapped with selected white matter tracts, including the CST, FMIN, and CGC. These tracts were selected based on the extent and presumed importance of their overlap with symptom-related lesions.

## 4 Discussion

This study investigated white matter tract involvement in post-stroke CRPS, focusing exclusively on patients with subcortical stroke. The diagnosis of CRPS was confirmed using a three-phase bone scan, ensuring objective verification of the condition. Among various major white matter tracts, only the CGC showed a statistically significant overlap with CRPS-related lesions.

The cingulate cortex, a crucial limbic structure, integrates emotional and cognitive aspects of pain processing (20). It is consistently activated by noxious stimuli and exhibits functional plasticity in chronic pain states, such as CRPS, underscoring its central role in the affective dimension of pain perception and in modulating descending pain-inhibitory pathways (21). A meta-analysis identified gray matter volume loss extending to the right median cingulate gyrus (middle cingulate) and the adjacent corpus callosum, suggesting that cingulate atrophy is a reproducible feature of CRPS (22). Consistent with these findings, our study demonstrated greater cingulate cortex involvement in stroke patients with CRPS than those without, supporting its potential role in the pathophysiology of post-stroke CRPS.

Although CST involvement did not differ significantly between groups, shoulder flexor MMT scores were significantly lower in the CRPS group. This suggests that CST integrity alone does not fully explain the motor deficits observed in CRPS, implicating alternative or additional mechanisms in patients with subcortical stroke. Several such mechanisms have been previously identified. One study demonstrated that cortical reorganization and disrupted integration of sensory feedback contribute to CRPS-related motor deficits through altered sensorimotor processing and body schema distortion (23). Another study suggested that pain-induced motor adaptations may lead to incomplete muscle recruitment, resulting in apparent weakness (24). Furthermore, dissociation of the affected limb, often described as a neglect-like symptom, has been recognized as a distinctive feature of CRPS, highlighting the role of cognitive and psychological mechanisms in motor impairment (25). These findings underscore the multifactorial origins of motor weakness in CRPS and emphasize the importance of understanding these diverse mechanisms to improve clinical assessment and rehabilitation strategies.

Early recognition and prompt intervention are essential to achieve favorable outcomes in post-stroke CRPS, as delayed treatment may result in persistent pain, joint contractures, and functional decline (26). Effective management requires a multidisciplinary approach integrating rehabilitative interventions with targeted pain control strategies. Physical therapy modalities, such as graded motor imagery and mirror therapy, play a vital role in maintaining limb function, reducing pain, and mitigating secondary complications (27). Pharmacological management typically addresses both neuropathic pain and inflammatory processes with commonly employed agents, including gabapentinoids, corticosteroids, and bisphosphonates (28). In addition, neuromodulation techniques such as dorsal root ganglion stimulation and spinal cord stimulation have also shown favorable effects (29). A meta-analysis demonstrated that repetitive transcranial magnetic stimulation yielded significant pain reduction, with effects emerging 1 week after the completion of the stimulation protocol (30). In line with this, a recent case report demonstrated that stimulation targeting the anterior cingulate cortex produced marked symptom improvement in CRPS (31). Together with our findings implicating the cingulate cortex in post-stroke CRPS, these results suggest this region as a potential therapeutic target for future neuromodulatory interventions.

This study had some limitations. First, the relatively small sample size may limit the generalizability of our findings. Small samples in voxel-based lesion-symptom analysis increase the risk of false negative results, which may be further amplified by FDR correction (32, 33). Therefore, the negative findings for tracts other than the CGC should be interpreted with caution. Moreover, although the CRPS group showed lower mean FMA and MBI scores, these differences were not statistically significant, possibly due to the limited sample size. Therefore, the present findings should be interpreted with caution, and future studies with larger sample sizes are warranted to clarify these associations more robustly. Second, although a significant difference in lesion involvement in CGC was identified between groups, this did not translate into a statistically significant association with functional outcomes. Third, both infarction and hemorrhage cases were included in the analysis, which may have introduced heterogeneity in lesion imaging characteristics and temporal evolution (34). However, previous studies have also included both hemorrhagic and ischemic strokes due to sample size limitations and have nevertheless provided meaningful and reproducible results (35–37). Nonetheless, although subgroup analyses by stroke subtype revealed no statistically significant differences within either subgroup, there was a trend toward greater CGC involvement in hemorrhagic cases compared to infarction, warranting further investigation in future studies. Fourth, we investigated lesion-induced white matter damage without diffusion tensor imaging (DTI), which limits our ability to interpret our findings. Consequently, our findings cannot substitute for direct assessments of tract integrity or connectivity, such as connectome-based lesion-symptom mapping that incorporates structural connectivity information (38). Findings from atlas-based overlap analyses should therefore be distinguished from direct tract integrity measures. Beyond the volumetric white matter atlases used here, recent studies utilizing population-based normative connectomes have been suggested (39, 40). Future studies employing such normative connectome analyses may offer a more comprehensive characterization of the impact of focal lesions on white matter networks. Finally, the retrospective design of this study limits causal inferences. Future prospective studies with more detailed standardized assessments are warranted to validate and expand upon these findings.

## Data Availability

The raw data supporting the conclusions of this article will be made available by the authors, without undue reservation.

## References

[1] Su YC Guo YH Hsieh PC Lin YC. A meta-analysis and meta-regression of frequency and risk factors for poststroke complex regional pain syndrome. Medicina. (2021) 57:57. doi: 10.3390/medicina57111232, PMID: 34833449 PMC8622266

[2] Kim CY Choi SB Lee ES. Prevalence and predisposing factors of post-stroke complex regional pain syndrome: retrospective case-control study. J Stroke Cerebrovasc Dis. (2024) 33:107522. doi: 10.1016/j.jstrokecerebrovasdis.2023.107522, PMID: 38141321

[3] Lee JI Kwon SW Lee A Tae WS Pyun SB. Neuroanatomical correlates of poststroke complex regional pain syndrome: a voxel-based lesion symptom-mapping study. Sci Rep. (2021) 11:13093. doi: 10.1038/s41598-021-92564-7, PMID: 34158602 PMC8219671

[4] Pellicciari MC Bonnì S Ponzo V Cinnera AM Mancini M Casula EP . Dynamic reorganization of TMS-evoked activity in subcortical stroke patients. NeuroImage. (2018) 175:365–78. doi: 10.1016/j.neuroimage.2018.04.011, PMID: 29635028

[5] Thickbroom GW Byrnes ML Archer SA Mastaglia FL. Motor outcome after subcortical stroke correlates with the degree of cortical reorganization. Clin Neurophysiol. (2004) 115:2144–50. doi: 10.1016/j.clinph.2004.04.001, PMID: 15294217

[6] Cha S Jeong B Choi M Kwon S Lee SH Paik NJ . White matter tracts involved in subcortical unilateral spatial neglect in subacute stroke. Front Neurol. (2022) 13:992107. doi: 10.3389/fneur.2022.992107, PMID: 36247754 PMC9561922

[7] Friston KJ. Statistical parametric mapping In: Kötter R, editor. Neuroscience Databases. Boston, MA: Springer (2003). 237–50.

[8] Rorden C Bonilha L Fridriksson J Bender B Karnath H-O. Age-specific CT and MRI templates for spatial normalization. NeuroImage. (2012) 61:957–65. doi: 10.1016/j.neuroimage.2012.03.020, PMID: 22440645 PMC3376197

[9] Rorden C Brett M. Stereotaxic display of brain lesions. Behav Neurol. (2000) 12:191–200. doi: 10.1155/2000/421719, PMID: 11568431

[10] Kim G Jeong B Choi M Kim WS Han CE Paik NJ. Neural substrates of subcortical aphasia in subacute stroke: voxel-based lesion symptom mapping study. J Neurol Sci. (2021) 420:117266. doi: 10.1016/j.jns.2020.117266, PMID: 33341084

[11] Hua K Zhang J Wakana S Jiang H Li X Reich DS . Tract probability maps in stereotaxic spaces: analyses of white matter anatomy and tract-specific quantification. NeuroImage. (2008) 39:336–47. doi: 10.1016/j.neuroimage.2007.07.053, PMID: 17931890 PMC2724595

[12] Mori S Wakana S Nagae-Poetscher L Van Zijl P. Mri atlas of human white matter. AJNR Am J Neuroradiol. (2006) 27:1384.

[13] Cho EB Han CE Seo SW Chin J Shin J-H Cho H-J . White matter network disruption and cognitive dysfunction in neuromyelitis optica spectrum disorder. Front Neurol. (2018) 9:1104. doi: 10.3389/fneur.2018.01104, PMID: 30619061 PMC6304415

[14] Nichols T Ridgway G Webster M Smith S. (2008). GLM permutation-nonparametric inference for arbitrary general linear models. Available online at: https://discovery.ucl.ac.uk/id/eprint/13848/1/13848.pdf (Accessed October 24, 2025).

[15] Genovese CR Lazar NA Nichols T. Thresholding of statistical maps in functional neuroimaging using the false discovery rate. NeuroImage. (2002) 15:870–8. doi: 10.1006/nimg.2001.1037, PMID: 11906227

[16] Benjamini Y Hochberg Y. Controlling the false discovery rate: a practical and powerful approach to multiple testing. J R Stat Soc B. (1995) 57:289–300. doi: 10.1111/j.2517-6161.1995.tb02031.x

[17] Mulchrone KF. Linstat, a program for calculating finite strain from populations of lines, running simulations and an investigation of error behaviour. Comput Geosci. (2003) 29:639–46. doi: 10.1016/S0098-3004(03)00046-3

[18] Jenkinson M Beckmann CF Behrens TEJ Woolrich MW Smith SM. FSL. NeuroImage. (2012) 62:782–90. doi: 10.1016/j.neuroimage.2011.09.015 21979382

[19] Xia M Wang J He Y. BrainNet viewer: a network visualization tool for human brain connectomics. PLoS One. (2013) 8:e68910. doi: 10.1371/journal.pone.0068910, PMID: 23861951 PMC3701683

[20] Franciosa F Acuña MA Nevian NE Nevian T. A cellular mechanism contributing to pain-induced analgesia. Pain. (2024) 165:2517–29. doi: 10.1097/j.pain.0000000000003315, PMID: 38968393 PMC11474934

[21] Freund W Wunderlich AP Stuber G Mayer F Steffen P Mentzel M . The role of periaqueductal gray and cingulate cortex during suppression of pain in complex regional pain syndrome. Clin J Pain. (2011) 27:796–804. doi: 10.1097/AJP.0b013e31821d9063, PMID: 21593662

[22] Ma T Li ZY Yu Y Yang Y Ni MH Xie H . Gray matter abnormalities in patients with complex regional pain syndrome: a systematic review and meta-analysis of voxel-based morphometry studies. Brain Sci. (2022) 12:12. doi: 10.3390/brainsci12081115, PMID: 36009176 PMC9405829

[23] Bank PJM Peper CLE Marinus J Beek PJ van Hilten JJ. Motor dysfunction of complex regional pain syndrome is related to impaired central processing of proprioceptive information. J Pain. (2013) 14:1460–74. doi: 10.1016/j.jpain.2013.07.009 24064035

[24] Bank PJM Peper CLE Marinus J Beek PJ van Hilten JJ. Deficient muscle activation in patients with complex regional pain syndrome and abnormal hand postures: an electromyographic evaluation. Clin Neurophysiol. (2013) 124:2025–35. doi: 10.1016/j.clinph.2013.03.029 23692976

[25] Frettlöh J Hüppe M Maier C. Severity and specificity of neglect-like symptoms in patients with complex regional pain syndrome (CRPS) compared to chronic limb pain of other origins. Pain. (2006) 124:184–9. doi: 10.1016/j.pain.2006.04.010, PMID: 16730904

[26] Chae J. Poststroke complex regional pain syndrome. Top Stroke Rehabil. (2010) 17:151–62. doi: 10.1310/tsr1703-151, PMID: 20797958

[27] Donati D Boccolari P Giorgi F Berti L Platano D Tedeschi R. Breaking the cycle of pain: the role of graded motor imagery and mirror therapy in complex regional pain syndrome. Biomedicine. (2024) 12:2140. doi: 10.3390/biomedicines12092140, PMID: 39335652 PMC11428672

[28] Lima Pessôa B Netto JGM Adolphsson L Longo L Hauwanga WN McBenedict B. Complex regional pain syndrome: diagnosis, pathophysiology, and treatment approaches. Cureus. (2024) 16:e76324. doi: 10.7759/cureus.76324, PMID: 39850174 PMC11756781

[29] Samuel EA Ahmad K Manongi NJ Rajapandian R Moti Wala S AlEdani EM . The efficacy of neuromodulation, interventional treatment and unconventional therapies in the treatment of complex regional pain syndrome: a systematic review. Cureus. (2024) 16:e74248. doi: 10.7759/cureus.74248, PMID: 39712760 PMC11663435

[30] Chang MC Kwak SG Park D. The effect of RTMS in the management of pain associated with CRPS. Transl Neurosci. (2020) 11:363–70. doi: 10.1515/tnsci-2020-0120, PMID: 33335776 PMC7711855

[31] Doshi PP Sheth T Patole S Doshi PK. Use of deep repetitive transcranial magnetic (DTMS) stimulation of motor cortex and anterior cingulate cortex for complex regional pain syndrome type II: a case report. Transcranial Magn Stimul. (2025) 4:100171. doi: 10.1016/j.transm.2025.100171

[32] Lorca-Puls DL Gajardo-Vidal A White J Seghier ML Leff AP Green DW . The impact of sample size on the reproducibility of voxel-based lesion-deficit mappings. Neuropsychologia. (2018) 115:101–11. doi: 10.1016/j.neuropsychologia.2018.03.014, PMID: 29550526 PMC6018568

[33] Mirman D Landrigan JF Kokolis S Verillo S Ferrara C Pustina D. Corrections for multiple comparisons in voxel-based lesion-symptom mapping. Neuropsychologia. (2018) 115:112–23. doi: 10.1016/j.neuropsychologia.2017.08.025, PMID: 28847712 PMC5826816

[34] Kakkar P Kakkar T Patankar T Saha S. Current approaches and advances in the imaging of stroke. Dis Model Mech. (2021) 14:dmm048785. doi: 10.1242/dmm.048785, PMID: 34874055 PMC8669490

[35] Forkel SJ Catani M. Lesion mapping in acute stroke aphasia and its implications for recovery. Neuropsychologia. (2018) 115:88–100. doi: 10.1016/j.neuropsychologia.2018.03.036, PMID: 29605593 PMC6018610

[36] Frenkel-Toledo S Levin MF Berman S Liebermann DG Baniña MC Solomon JM . Shared and distinct voxel-based lesion-symptom mappings for spasticity and impaired movement in the hemiparetic upper limb. Sci Rep. (2022) 12:10169. doi: 10.1038/s41598-022-14359-8, PMID: 35715476 PMC9206020

[37] Khalilian M Roussel M Godefroy O Aarabi A. Predicting functional impairments with lesion-derived disconnectome mapping: validation in stroke patients with motor deficits. Eur J Neurosci. (2024) 59:3074–92. doi: 10.1111/ejn.16334, PMID: 38578844

[38] Gleichgerrcht E Fridriksson J Rorden C Bonilha L. Connectome-based lesion-symptom mapping (CLSM): a novel approach to map neurological function. NeuroImage Clin. (2017) 16:461–7. doi: 10.1016/j.nicl.2017.08.018, PMID: 28884073 PMC5581860

[39] Yeh FC Panesar S Fernandes D Meola A Yoshino M Fernandez-Miranda JC . Population-averaged atlas of the macroscale human structural connectome and its network topology. NeuroImage. (2018) 178:57–68. doi: 10.1016/j.neuroimage.2018.05.027, PMID: 29758339 PMC6921501

[40] Griffis JC Metcalf NV Corbetta M Shulman GL. Structural disconnections explain brain network dysfunction after stroke. Cell Rep. (2019) 28:2527–2540.e9. doi: 10.1016/j.celrep.2019.07.100, PMID: 31484066 PMC7032047

