# Supplementary material for: White matter tracts involved in complex regional pain syndrome after subcortical stroke
Source: Front Neurol. 2025 Nov 24;16:1699775. doi: 10.3389/fneur.2025.1699775 (PMC12682577; doi:10.3389/fneur.2025.1699775)
Supplement: Supplementary file 1 [file Table_1.DOCX]

**Supplementary Material**

**Supplementary Table 1.** Overlap (mm^3^) of the lesions with the major white matter tracts in patients with infarction only (n = 42)

| White matter tractography atlas | CRPS | Control | F-statistics | Uncorrected P-value | FDR-adjusted P-value |
| --- | --- | --- | --- | --- | --- |
| Anterior thalamic radiation | 32[0…330] | 48[0…1294] | 1.657 | 0.21 | 0.97 |
| Corticospinal tract | 168[0…698] | 360[4…582] | 0.041 | 0.84 | 0.97 |
| Cingulum (cingulate gyrus) | 0[0…0] | 0[0…0] | 1.107 | 0.36 | 0.97 |
| Cingulum (hippocampus) | 0[0…0] | 0[0…0] | n/a | n/a | n/a |
| Forceps major | 0[0…0] | 0[0…0] | 0.689 | 0.64 | 0.97 |
| Forceps minor | 0[0…0] | 0[0…0] | 0.631 | 0.71 | 0.97 |
| Inferior fronto-occipital fasciculus | 0[0…160] | 136[0…582] | 1.525 | 0.23 | 0.97 |
| Inferior longitudinal fasciculus | 0[0…0] | 0[0…0] | 0.016 | 0.88 | 0.97 |
| Superior longitudinal fasciculus | 0[0…338] | 0[0…12] | 0.197 | 0.76 | 0.97 |
| Uncinate fasciculus | 0[0…0] | 0[0…18] | 1.154 | 0.30 | 0.97 |
| Superior longitudinal fasciculus | 0[0…0] | 0[0…0] | 0.514 | 0.73 | 0.97 |

**Abbreviations**: CRPS, complex regional pain syndrome; FDR, false discovery rate.

**Supplementary Table 2.** Overlap (mm^3^) of the lesions with the major white matter tracts in patients with hemorrhage only (n = 38)

| White matter tractography atlas | CRPS | Control | F-statistics | Uncorrected P-value | FDR-adjusted P-value |
| --- | --- | --- | --- | --- | --- |
| Anterior thalamic radiation | 992[218…3174] | 64[0…376] | 5.313 | **0.027*** | 0.22 |
| Corticospinal tract | 1152[696…1894] | 1232[782…2126] | 0.019 | 0.89 | 0.89 |
| Cingulum (cingulate gyrus) | 0[0…124] | 0[0…0] | 3.395 | 0.05 | 0.22 |
| Cingulum (hippocampus) | 0[0…0] | 0[0…0] | 0.856 | 0.50 | 0.70 |
| Forceps major | 0[0…0] | 0[0…0] | 1.145 | 0.40 | 0.70 |
| Forceps minor | 0[0…574] | 0[0…0] | 3.308 | 0.06 | 0.22 |
| Inferior fronto-occipital fasciculus | 2016[1296…2594] | 1088[566…1922] | 1.975 | 0.17 | 0.46 |
| Inferior longitudinal fasciculus | 440[16…1498] | 64[0…1486] | 0.210 | 0.65 | 0.71 |
| Superior longitudinal fasciculus | 800[0…3030] | 480[0…3198] | 0.296 | 0.59 | 0.71 |
| Uncinate fasciculus | 400[60…458] | 96[0…492] | 1.291 | 0.26 | 0.58 |
| Superior longitudinal fasciculus | 0[0…0] | 0[0…0] | 0.856 | 0.51 | 0.70 |

*P-value < 0.05

**Abbreviations**: CRPS, complex regional pain syndrome; FDR, false discovery rate.
